# Spread of Amphibian Chytrid Fungus across Lowland Populations of Túngara Frogs in Panamá

**DOI:** 10.1371/journal.pone.0155745

**Published:** 2016-05-13

**Authors:** Sofía Rodríguez-Brenes, David Rodriguez, Roberto Ibáñez, Michael J. Ryan

**Affiliations:** 1 Department of Integrative Biology, University of Texas, Austin, Texas, United States of America; 2 Department of Biology, Texas State University, San Marcos, Texas, United States of America; 3 Smithsonian Tropical Research Institute, Balboa, Panamá; 4 Departamento de Zoología, Universidad de Panamá, Panamá, Panamá; Vanderbilt University School of Medicine, UNITED STATES

## Abstract

Chytridiomycosis, caused by the fungal pathogen *Batrachochytrium dendrobatidis* (*Bd*), is an emergent infectious disease partially responsible for worldwide amphibian population declines. The spread of *Bd* along highland habitats (> 500 meters above sea level, m a.s.l.) of Costa Rica and Panamá is well documented and has been linked to amphibian population collapses. In contrast, data are scarce on the prevalence and dispersal of *Bd* in lowland habitats where amphibians may be infected but asymptomatic. Here we describe the spread (2009 to 2014) of *Bd* across lowland habitats east of the Panamá Canal (< 500 m a.s.l.) with a focus on the Túngara frog (*Physalaemus [Engystomops] pustulosus*), one of the most common and abundant frog species in this region. Highland populations in western Panamá were already infected with *Bd* at the start of the study, which was consistent with previous studies indicating that *Bd* is enzootic in this region. In central Panamá, we collected the first positive samples in 2010, and by 2014, we detected *Bd* from remote sites in eastern Panamá (Darién National Park). We discuss the importance of studying *Bd* in lowland species, which may serve as potential reservoirs and agents of dispersal of *Bd* to highland species that are more susceptible to chytridiomycosis.

## Introduction

Wildlife extinctions are not typically attributed to infectious diseases, yet there are a few examples showing pathogens as the direct cause of local extinctions [1–3]. Some of them have been linked to chytridiomycosis, an emerging infectious disease [4,5]. In amphibians, chytridiomycosis results from a skin infection caused by the chytrid fungus *Batrachochytrium dendrobatidis* (*Bd*). In the tropics, the most severe declines have been documented in comparatively cooler and humid areas above 500 m [6], most likely because lower temperatures (17–25°C) are optimal for *Bd* growth and water facilitates the propagation and dispersal of the aquatic, flagellated *Bd* zoospores [7]. Although the rapid spread of *Bd* into apparently *Bd*-free areas throughout the world is well-documented [8–12], the process by which *Bd* spreads is still not clearly understood [13].

**Fig 1:**
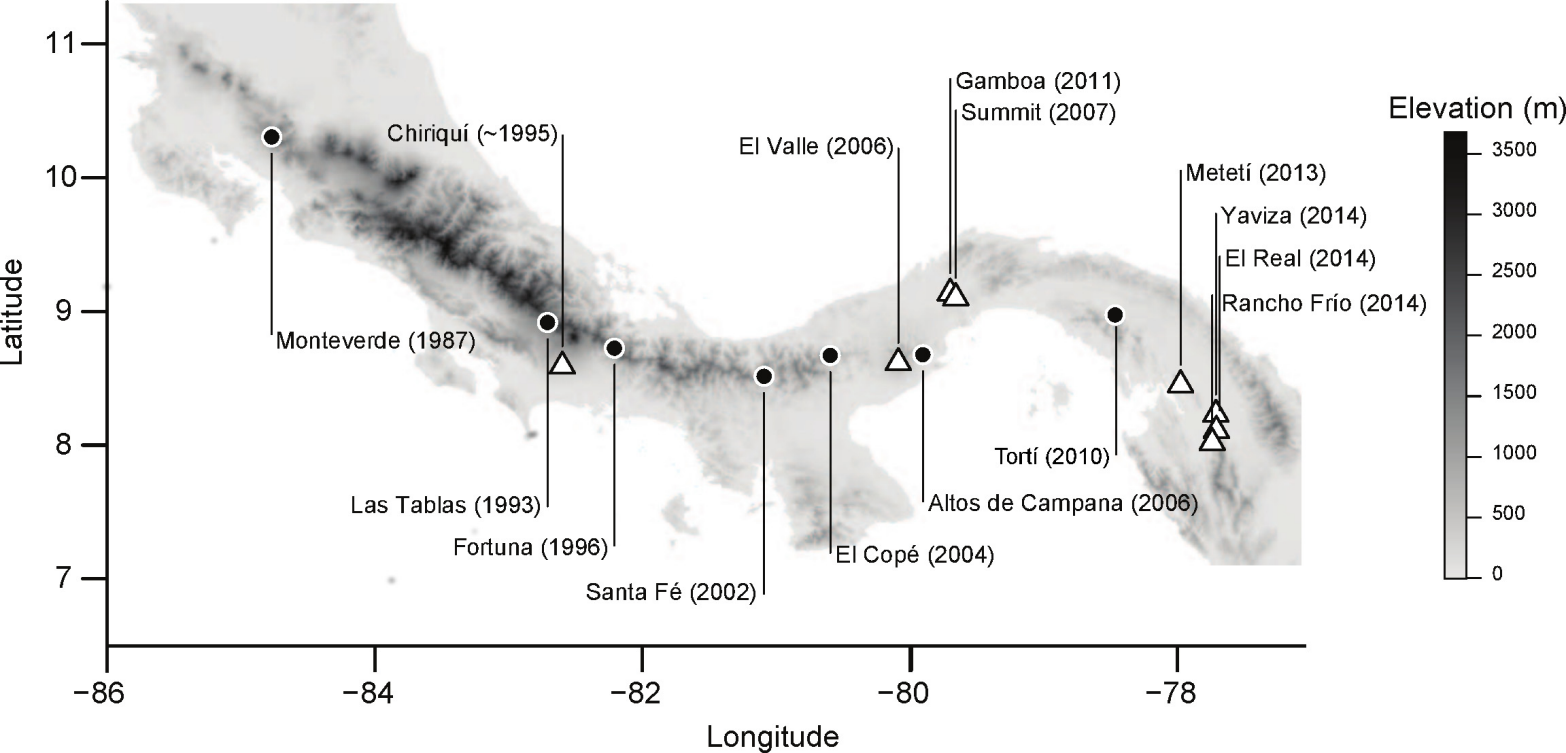
Sites where *Bd* has been detected in the past (filled circles), and sites where we sampled Túngara frog *(Physalaemus [Engystomops] pustulosus)* populations (open triangles). Year in parenthesis corresponds to year of decline or the year that *Bd* was first detected.

*Bd* has rapidly spread throughout the highlands of Central America. In the late 1980s, the disappearance of golden toads (*Incilius periglenes*) and declines of other anuran populations in the protected cloud forest of Monteverde, Costa Rica (Fig 1), were the first alerts to what later became a predictable pattern of declines spreading towards Panamá [14,15]. Shortly thereafter, between 1993 and 1997 additional cases of amphibian population declines or extinctions were reported in the highlands close to the border with Panamá at Las Tablas, Costa Rica, and Fortuna, Panamá (Fig 1)[16,17]. The sites experiencing population declines were located at altitudes above 500 m, and the fastest declining species were stream dwellers with aquatic tadpoles [6]. *Bd* was spreading west to east in a wave like pattern [8]. High elevation sites in western and central Panamá were then intensively studied prior to and during the arrival of *Bd*, these studies documented amphibian population declines after the arrival of *Bd* in this region (Fig 1)[16–19].

Only two studies have investigated *Bd* infection spread in eastern Panamá. Rebollar et al. [20] sampled populations along the lowlands of east Panamá, and Woodhams et al. [19] sampled sites in central Panamá. These studies did not detect a clear pattern of wave-like spread from west to east as observed in the highlands of Panamá. It is also possible that another wave of *Bd* from South America crossed into Panamá [8]. Currently, the dynamics of *Bd* spread in the lowlands of Panamá, where environmental conditions are not ideal for *Bd*, are still unclear and require additional investigation.

Evaluating the spread of a pathogen in a single, abundant, and well-characterized host can help to predict spread dynamics while controlling for host phylogenetic diversity. The Túngara frog (*Physalaemus [Engystomops] pustulosus*) is a common species occupying lowland habitats ranging from Mexico to Colombia, Venezuela, and a small portion of the Guyana Shield. Its range parallels many highland regions where *Bd* and amphibian declines have been extensively documented [8–13]. The adults and tadpoles of this species use both permanent and ephemeral bodies of water in habitats ranging from urban areas to pristine forests. Thus, the Túngara frog is an ideal species in which to characterize the spread of *Bd* through populations of a lowland host. Here, we (i) report the *Bd* infection of túngara frogs at two sites west of the Panamá Canal where *Bd* is enzootic, and (ii) present data documenting the spread of *Bd* in the lowlands of central and east Panamá from 2009 to 2014.

## Methods

We sampled Túngara frog populations in Panamá from 2009 to 2014, each year during the rainy season (June to November), which falls within their reproductive season. In most cases, populations were sampled during two reproductive seasons. Lowland sites in east and central Panamá ranged from 12 to 67 m in altitude (Fig 1). We also sampled highland populations at El Valle (elevation 600 m; Fig 1) in central Panamá, and at Chiriquí near the town of Cuesta de Piedra (elevation 460–967 m; Fig 1) in western Panamá. This altitude is the highest known Túngara population in Panamá. Chiriquí was also our western most site and was located 30 km east of sites where other amphibian species experienced declines in 1993 [17].

In central Panamá, we sampled two lowland sites east of the Panamá Canal, Gamboa and Summit, ranging from 46 to 98 m in elevation (Fig 1). These two lowland sites are located on the eastern side of the Panamá Canal but are separated by the Chagres River. Gamboa is a small town north of the river that is surrounded by rainforest. Here we included Pipeline Road, which intersects a protected rainforest in the Soberanía National Park. Summit is located south of the river and includes portions of the Soberanía National Park. We sampled along the main road, on trails and dirt roads within the National Park.

In east Panamá, we sampled four sites. Metetí and Yaviza are located along and at the very end of the Inter-American Highway, respectively (Fig 1). Here, we sampled disturbed, deforested habitats, in puddles on dirt roads and cattle ranches. Further east, along the Tuira River, we sampled at El Real (Fig 1), a small town surrounded by less disturbed habitat and in Darién National Park. In Darién National Park, we sampled around the Rancho Frío field station (Fig 1) at an average of 50 m of elevation. This site is predominantly primary rainforest with a tall canopy, thick lianas and an understory dominated by palms.

### Time scale of surveys

We first sampled El Valle in 2009 and then Chiriquí in 2010, where *Bd* is thought to have arrived in the mid-1990s according to previous estimations [13]. In 2010, we also sampled Gamboa and Summit in central Panamá. Woodhams et al. [19] reported *Bd* in Summit in 2007. We then sampled Metetí and Yaviza in eastern Panamá during 2011, where we expected the front of the *Bd* wave to be just arriving. In 2013, we sampled all the populations previously surveyed. In 2014 we sampled in Gamboa, Yaviza and finally Darién National Park, which was the eastern most site and where we expected *P*. *pustulosus* populations to be *Bd* naive.

### *Bd* sampling

We toe-clipped individuals to avoid recapture. To avoid cross contamination, we captured adults by hand using a new pair of nitrile gloves for each individual and kept them isolated in a plastic bag until processing. We swabbed the ventral area using a sterile cotton tip dry swab (Medical Wire & Equipment, model MW113 and MW110) following established procedures [21]. Swabs were stored in 90% ethanol or they were kept frozen until extraction, thus we expect no effects of sample storage on estimates of prevalence or infection intensity [22]. To avoid potential cross-contamination between sites, we bleached our rubber boots and vehicle tires and then rinsed them with tap water before leaving the collection site.

### Real time quantitative PCR

To determine the prevalence of *Bd* in each population and the intensity of infection for individual frogs, we processed the swabs using quantitative PCR (qPCR) following the protocol developed by Boyle et al. [23] and modified by Kriger et al. [24]. For samples collected from 2009 to 2012, we used a Roche LightCycler 480 system with a high confidence setting to detect positive samples. For samples collected during 2009–2010, we did not quantify the number of zoospores due to lack of *Bd* standards. For samples collected during 2013 and 2014, we used TaqMan® Fast Advanced Master Mix (Applied Biosystems) and a StepOnePlus™ system. We used a dilution series of genomic DNA from strain JEL423 as our standard reference for the estimations of infection intensity in samples collected from 2011 to 2014. We calculated prevalence by using the ratio of positive samples to total samples per population and calculated 95% binomial confidence intervals. For each population, we calculated average infection intensity using the average number of zoospore equivalents (z.e.) inferred from qPCR among positive individuals.

To calculate the rate of *Bd* spread across sites east of the Canal, we used the distance between sites and the year we first detected infected frogs. We sampled frogs from June to November. If *Bd* arrived after sampling was completed for a given year, then we would have detected it the following year. We could not calculate the rate of spread in western Panamá because *Bd* was already present at the beginning of our sampling period. Thus, we approximated when Túngara frogs were first infected based on historical reports for sites near Chiriquí [8,13,19].

### Ethical approval

All applicable institutional and/or national guidelines for the care and use of animals were followed. The protocol was approved by the Institutional Animal Care and Use Committee of the Smithsonian Tropical Research Institute (protocol number: 2011-0825-2014-02), and by the Autoridad Nacional del Ambiente (permit numbers: SE/A-81-09, SE/A-73-10, SE/A-48-10, SC/A-28-11, SE/A-83-11, SE/A-42-11, SE/A-30-12, SE/A-47-13, SC/A-9-14)

## Results

In total, we sampled 1695 *P*. *pustulosus* adults across Panamá from 2009 to 2014 (Fig 1). Sample size, prevalence, and 95% binomial confidence intervals are shown in Table 1. We considered sites with sample sizes greater than 60 individuals and no positive samples as *Bd*-negative [25]. The site Chiriquí, in western Panamá, was positive for *Bd* in 2010 and 2013. In central Panamá, El Valle was positive for *Bd* in 2009. In 2010, Gamboa samples were negative for *Bd*, but Summit, which is only 8 km to the south, was positive. *Bd* reached Gamboa in 2011, and in following years we detected *Bd* positive samples in both Summit and Gamboa. By 2014, prevalence had reached 26% in Gamboa (Table 1). Populations in Metetí were *Bd* naive in 2011 but positive in 2013. Farther east, Yaviza was naive for *Bd* in 2013, but by 2014 prevalence reached approximately 6%. By 2014, *Bd* was also present in *P*. *pustulosus* populations from El Real and Rancho Frío, Darién National Park (Table 1).

**Table 1:** *Bd* prevalence and infection intensity per site and year in Túngara frog (*Physalaemus [Engystomops] pustulosus*) populations sampled in this study (n = number of individuals sampled; Positive = number of individuals detected positive for *Bd*; 95% CI = 95% binomial distribution confidence intervals; Average intensity = average of number of zoospore equivalents in infected frogs per population). Sites are arranged west to east by longitude.

Site	Year	N	Positive	Prevalence % (95% CI)	Average Intensity (± St.Dev)
Chiriquí	2010	41	11	27 (14–43)	data not available
	2013	38	16	42 (26–60)	3570 (± 12454)
El Valle	2009	5	3	60 (15–95)	data not available
Gamboa	2010	321	0	0 (0–0.5)	0
	2011	111	7	6 (3–13)	86 (± 117)
	2012	205	26	13 (8–18)	1617 (± 5485)
	2013	166	35	21 (15–29)	536 (± 2061)
	2014	84	22	26 (17–37)	209 (± 833)
Summit	2010	12	2	17 (2–48)	data not available
	2011	108	2	2 (0–6)	10 (± 7)
	2013	120	17	14 (9–22)	89 (± 193)
Metetí	2011	91	0	0 (0–4)	0
	2013	94	2	2 (0–8)	7 (± 3)
Yaviza	2013	68	0	0 (0–0.5)	0
	2014	41	3	7 (2–20)	6 (± 1)
El Real	2014	40	2	5 (1–17)	6 (± 1)
Rancho Frío	2014	150	11	7 (4–13)	48 (± 101)

### Rates of spread of *Bd* in Túngara frogs

If *Bd* spread eastward from Summit in central Panamá to Darién in eastern Panamá, then the front moved at an average rate of 54 km/year among these lowland Túngara frog populations. The rates of spread, however, vary substantially. Specifically, our data suggest that it took approximately one year for *Bd* to move the 8 km distance between Summit and Gamboa, which are separated by the Chagres River. In contrast, the rate of *Bd* spread from Summit to Metetí was 65 km/year, and 42 km/year from Metetí to Yaviza.

All data are available from the DRYAD Digital Repository (doi:10.5061/dryad.6bp92).

## Discussion

Data for the spread of *Bd* in the lowlands of Middle America are scarce. Here we estimated the rate of spread of *Bd* in Túngara frogs based on the first detection of *Bd*, and we assumed that *Bd* spread in a wave-like fashion [8]. In the lowlands of Panamá, *Bd* spread at a similar rate among Túngara frogs (54 km/year) when compared to other amphibian species tested in this area (30–174 km/year) [19]. There was one exception. The rate of spread from Summit across the Chagres River to Gamboa was slower than the average (8 km/year).

In western Panamá, we sampled in Chiriquí, at elevations where amphibian population declines have been severe in the past [16]. Since *Bd* is now enzootic in this area [19] and at El Valle in central Panamá, it is not surprising that the Túngara frog populations from Chiriquí and El Valle were positive in 2010, and 2009, respectively. We also found the highest average loads of *Bd* in Chiriquí (3570 z.e., Table 1). We could not estimate the rate of *Bd* dispersal among Túngara frog populations between Chiriquí and El Valle as they were already infected at the time we sampled. In 2007, Woodhams et al. [19] reported *Bd* positive individuals (30% prevalence) among three species at Soberanía National Park, including the Summit area where we sampled in 2010, and they suggested that *Bd* was already enzootic during their study. In the same year, Gamboa, was still naive and we most likely sampled before *Bd* arrived. In 2011, we detected *Bd* at Gamboa for the first time, one year earlier than previously reported for this area [20].

We cannot discern whether the source of infection of the Gamboa populations was from Summit Túngara frogs or from other species of frogs in Gamboa. Regardless, there is a relatively large time lag between when *Bd* was detected in Túngara frogs from Gamboa compared to when *Bd* was first detected in Túngara frogs from Summit. The mechanism by which *Bd* spreads is unknown, but it has been suggested that *Bd* could survive and could be carried in mud, and thus easily dispersed by humans [26]. In Túngara frogs, *Bd* did not spread as fast as expected from Summit to Gamboa, which are only 8 km apart and connected by a well-traveled road and bridge over the Chagres River.

Certain geographic features, like rivers, could impede the spread of *Bd*. The Chagres River, which is about 100 m wide and has water all year long, separates Gamboa and Summit. Genetic studies demonstrate that the Chagres River is a geographical barrier for gene flow between Túngara frog populations [27], thus it is possible that limited migration between these populations could slow the spread of *Bd* whether Túngara frogs contracted *Bd* from conspecifics or heterospecifics. *Bd* does seem to have spread rapidly in the lowlands towards eastern Panamá. An alternative explanation for the spread of *Bd* from east to west throughout all of Panamá is that populations in Darién, and elsewhere in far eastern Panamá, were infected by a wave coming from the south [8]. These two scenarios should be tested through a phylogenetic analysis of *Bd* throughout the Túngara frog's range in Central and South America. As such data are currently unavailable, it is difficult to determine the recent origin of *Bd* in eastern Panamá; therefore, our estimates of rate of spread should be viewed with this potential caveat in mind.

Woodhams *et al*. [19] conservatively estimated that *Bd* spread east and would have reached Tortí in September of 2012, but [28] reported two positives out of 93 samples of other species of frogs at this site in 2010. There are no other published reports on the presence of *Bd* among amphibian species from this specific area. In 2011, we sampled in Metetí, approximately 70 km southeast of Tortí, and found this site to be *Bd* naive. Rebollar *et al*. [20] recorded *Bd* positive samples from Nuevo Vigía in 2012, just 26 km east of Metetí, where we detected low *Bd* prevalence in 2013, thus supporting a wave-like *Bd* spread from west to east in Túngara frogs.

Species that carry *Bd* asymptomatically and share the habitat with more vulnerable species can potentially function as *Bd* spreaders. Túngara frog population declines have not been reported and were not evident during our study. If the prevalence and dispersal of *Bd* is density dependent, then the spread of *Bd* along the lowlands might be enhanced by abundant and apparently resistant species serving as reservoirs. Túngara frogs are known to disperse between breeding sites at distances up to 200 m [29]. They also share the habitat with a wide variety of species; thus, they could contribute to the rapid dispersal of *Bd* if they are effective carriers. Moreover, if most species in the lowlands are less susceptible to *Bd* or *Bd* is less virulent [7], then the high diversity and abundance of hosts could further facilitate the dispersal of *Bd*.

Chytridiomycosis is linked to some of the most severe population declines and extinctions of wildlife yet recorded. While substantial efforts have been aimed at understanding the spread and pathogenicity in the more vulnerable highland frog species, we know relatively little about the dynamics of *Bd* in tropical lowland regions of the world. Lowland species, however, could be reservoirs and dispersal agents between areas where amphibian species are more vulnerable to *Bd* infection. As highlighted by our results, even though lowland regions are typically characterized by less favorable climatic conditions for *Bd* [7], by harboring asymptomatic *B*d infections, lowland amphibian populations could potentially play an important role in the spread of *Bd* across tropical regions.
